# Special Issue “Advanced Nanomaterials for Bioimaging”

**DOI:** 10.3390/nano12142496

**Published:** 2022-07-20

**Authors:** Gang Ho Lee

**Affiliations:** Department of Chemistry, College of Natural Sciences, Kyungpook National University, Taegu 41566, Korea; ghlee@mail.knu.ac.kr; Tel.: +82-53-950-5340

Bioimaging currently plays a critical role in medical diagnosis [1,2,3]. Various imaging modalities are available for this purpose, such as magnetic resonance imaging (MRI), X-ray computed tomography (CT), ultrasound imaging, fluorescence imaging (FI), positron emission tomography (PET), and single-photon emission computed tomography (SPECT). Molecular imaging agents are a requisite for FI [4,5], PET [6], and SPECT [6], but they are also commonly used for MRI [7] and CT [8] in order to improve imaging sensitivity and resolution. Currently, most commercial imaging agents are molecular agents [6,7,8]. However, nanomaterials such as nanoparticles, nanorods, core-shell nanoparticles, and hybrid nanoparticles have been extensively investigated as imaging agents owing to their unique and advanced properties [9,10], which are highly advantageous for disease diagnosis and treatment [11,12].

This Special Issue focuses on various nanomaterials that are useful for imaging, drug delivery, and therapy. Nanoparticles possess several advantages over small molecules: they have advanced imaging properties, easy surface functionalization for tumor-targeted drug delivery, and longer blood circulation times. Nanoparticles also have higher metal ion concentrations than small molecules at the same number density, a property that makes them highly favorable CT contrast agents [13,14,15,16]. Gadolinium-containing nanoparticles exhibit higher longitudinal water proton spin relaxivities (r_1_) than Gd(III)-chelates do, and hence are useful high-performance T_1_ MRI contrast agents [10,11,13,14]. Fatima et al. reviewed recent advances in gadolinium-based contrast agents for bioimaging [17]. Gadolinium-based nanoparticles are useful as T_1_ MRI contrast agents because of the high and pure spin of Gd^3+^ (s = 7/2) and the high density of Gd^3+^ per nanoparticle; these lead to high r_1_ values, as well as r_2_/r_1_ ratios close to 1 (where r_2_ is the transverse water proton spin relaxivity). In addition, owing to the strong X-ray attenuation property of gadolinium, these nanoparticles are useful as CT contrast agents [13,14,15]. Dual T_1_ MRI-FI is also possible, by doping fluorescent europium or terbium metal ions into or conjugating dyes with gadolinium-based nanoparticles [17]. Holmium- and terbium-containing nanoparticles can be used as T_2_ MRI contrast agents owing to their appreciable magnetic moments at room temperature, a behavior that arises from the 4f-electron spin-orbital motions of these elements. Liu et al. prepared polyethylenimine (Mn = 1200 amu)-coated ultrasmall holmium oxide nanoparticles (average particle diameter: 2.05 nm) and measured their cytotoxicity and water proton spin relaxivities [18]. Notably, the nanoparticles exhibited an r_2_ value of 13.1 s^−1^ mM^−1^ with a negligible r_1_ value of 0.1 s^−1^ mM^−1^, which results in very large r_2_/r_1_ ratios and makes them efficient T_2_ MRI contrast agents. These nanoparticles exclusively induce T_2_ relaxations, with negligible induction of T_1_ relaxations, and can thus act as efficient T_2_ MRI contrast agents. Shanti et al. proved this phenomenon in vivo by successfully administering polyacrylic acid-coated terbium(III) and holmium(III) oxide nanoparticles to mice in 3.0 and 9.4 T MR fields [19]. They observed appreciable T_2_ contrasts in the livers and kidneys of the mice after injection, with enhanced T_2_ contrasts at 9.4 T.

FI is a highly sensitive bioimaging modality that is useful for cell imaging and skin-deep in vivo imaging due to its low penetration depth (<1 cm) [2,3]. Compared with organic dyes and quantum dots [4,5], terbium- and europium-containing nanomaterials possess larger Stokes shifts and show less background noise in biological samples; thus, they are extremely useful for disease detection at very low concentrations. Gómez-Morales and coworkers synthesized luminescent citrate-functionalized terbium-substituted carbonated apatite nanomaterials and applied them to cellular uptake imaging [20]. The luminescence properties of these nanoparticles allowed visualization of their intracellular cytoplasmic uptake after 12 h of treatment through flow cytometry and fluorescence confocal microscopy (green fluorescence was observed) when incubated with A375 cells.

Nanomaterials that combine imaging and therapy can be utilized in cancer theranostics. Such nanomaterials can be prepared by using nanoparticles with both imaging and therapeutic properties (such as gadolinium-containing nanomaterials [21]), or by synthesizing hybrid nanomaterials. Tian et al. prepared multifunctional magnetoplasmonic Au-MnO hybrid nanocomposites for cancer theranostics. MnO exhibited promising contrast enhancement in T_1_ MR imaging with good relaxivity (r_1_ = 1.2 mM^−1^ s^−1^), and Au produced sufficient heat (62 °C at 200 µg/mL) to ablate cancerous cells upon 808 nm laser irradiation (photothermal therapy), inducing cell toxicity and apoptosis [22].

Soft polymer nanomaterials are ideal drug delivery vehicles. They can also carry imaging materials to diagnose diseases. Zerrillo et al. synthesized poly(lactide-co-glycolide) (PLGA) nanoparticles and grafted them with hyaluronic acid (HA), which can bind to specific receptors in various cells, in order to improve site specificity and drug dose delivery in osteoarthritis nanotherapy [23]. They further encapsulated the nanoparticles with a near-infrared (NIR) dye and gold (20 nm). With the NIR dye and gold acting as contrast agents, the encapsulated HA-PLGA nanoparticles were successfully visualized on micro-CT by optical imaging in vivo in mouse knees and ex vivo in human cartilage explants.

This Special Issue covers a broad spectrum of nanomaterials that can be used for bioimaging. Surface modification of nanomaterials with hydrophilic ligands is essential for bioimaging applications, while further functionalization of surface-modified nanomaterials with drugs and cancer-targeting ligands will make them invaluable cancer-targeting theranostic agents.

## Data Availability

Data might be available from the authors of the cited papers.
